# Association between infertility and thyroid cancer risk: a systematic review and meta-analysis

**DOI:** 10.3389/fonc.2026.1813942

**Published:** 2026-05-18

**Authors:** Xianli Qiao, Xiangyang Tian, Wenya Zhou, Zemin Qin, Junyan Yu

**Affiliations:** Department of Oncology, Heping Hospital affiliated to Changzhi Medical College, Changzhi, China

**Keywords:** infertility, meta-analysis, risk factors, systematic review, thyroid cancer

## Abstract

**Background:**

Epidemiologic evidence on the association between infertility and thyroid cancer risk remains inconsistent. We conducted a systematic review and meta-analysis to synthesize available observational evidence and quantify this association.

**Methods:**

We systematically searched PubMed, Web of Science, and Scopus from inception to February 10, 2026, to identify observational studies evaluating the association between infertility and thyroid cancer risk. Eligible cohort and case–control studies reporting relative effect estimates with 95% confidence intervals (CIs) were included. Summary risk estimates were pooled using random-effects models. Between-study heterogeneity was assessed using the *I²* statistic. Prespecified subgroup analyses were conducted by sex and study design. Sensitivity analyses and assessments of publication bias were also performed.

**Results:**

Nine studies met the inclusion criteria, including seven cohort studies and two case–control studies, comprising a total of 4,563,090 participants. In the pooled analysis, infertility was associated with a significantly higher risk of thyroid cancer (relative risk [RR], 1.37; 95% CI, 1.15–1.63), with substantial heterogeneity across studies (*I²* = 81.9%). Sex-stratified analyses suggested a stronger association among men (RR, 1.53; 95% CI, 1.43–1.65) than among women (RR, 1.31; 95% CI, 1.04–1.66), although the test for interaction was not statistically significant. The findings were robust across sensitivity analyses. Evidence of small-study effects was observed; however, the association remained statistically significant after adjustment using the trim-and-fill method.

**Conclusions:**

This study suggests that infertility is associated with an increased risk of thyroid cancer. Although substantial heterogeneity was observed, the association remained consistent across multiple sensitivity analyses. However, further well-designed research is needed to verify and clarify this association and its underlying mechanisms.

## 1 Introduction

Thyroid cancer is one of the most common endocrine malignancies worldwide and disproportionately affects women, with incidence peaking during the reproductive years (1). Although ionizing radiation and genetic susceptibility are well-established risk factors, the sustained rise in thyroid cancer incidence over recent decades has renewed interest in the role of reproductive and endocrine influences (2–4). In parallel, infertility has become an increasingly prevalent public health issue, affecting an estimated 9–18% of couples globally, alongside expanding use of ovulation induction and assisted reproductive technologies (ART) (5). These converging trends have raised concern that infertility itself—and the hormonal disturbances inherent to both its underlying biology and treatment—may be associated with altered thyroid cancer risk (4, 6).

Biological plausibility for such an association is supported by shared endocrine pathways. Estrogen receptors are expressed in normal and malignant thyroid tissue, and experimental and clinical evidence suggests that estrogen signaling may promote thyroid cell proliferation and tumorigenesis (7, 8). Fertility treatments can also induce transient but substantial alterations in estrogen, progesterone, gonadotropins, and thyroid-stimulating hormone, potentially influencing thyroid growth and carcinogenic processes (9). Moreover, infertility frequently coexists with autoimmune and metabolic conditions that may independently modify thyroid cancer risk, complicating etiologic interpretation (10).

Despite these mechanistic considerations, epidemiologic evidence remains inconsistent. Some population-based cohort studies have reported higher thyroid cancer incidence among women with infertility. For instance, a large Taiwanese cohort observed an adjusted incidence rate ratio (IRR) of 1.80 overall, with a more pronounced excess after seven years of follow-up (IRR 4.39) (11). However, other observational studies have reported null or attenuated associations, underscoring substantial heterogeneity in findings (12–14).

Previous meta-analyses have largely focused on fertility treatments rather than infertility itself. A 2018 meta-analysis reported an increased thyroid cancer risk among infertile women exposed to fertility drugs, particularly clomiphene citrate (15), while more recent syntheses yielded similar findings but found no clear association for *in vitro* fertilization or ART (16). These analyses, however, often conflate infertility diagnoses with pharmacologic or procedural exposures, limiting inference regarding the independent role of infertility.

Given the growing prevalence of infertility and the rising global burden of thyroid cancer, a focused synthesis is warranted. Accordingly, we conducted a systematic review and meta-analysis of observational studies to quantify the association between infertility and thyroid cancer risk, explore potential sex-specific differences, and assess between-study heterogeneity.

## 2 Methods

### 2.1 Study design and eligibility criteria

This systematic review and meta-analysis were conducted following the Preferred Reporting Items for Systematic Reviews and Meta-Analyses (PRISMA) guidelines (17). The study focused on observational evidence examining the association between infertility and thyroid cancer risk, structured according to the PICOS framework: (1) Population (P): Adults of reproductive age (both men and women) with infertility, defined according to study-specific criteria, including clinical diagnosis, self-reported infertility, or documented fertility treatment. (2) Exposure (E): Infertility or fertility-related exposures, including primary or secondary infertility, regardless of etiology. (3) Comparator (C): Individuals without infertility or those not exposed to fertility-related factors. (4) Outcome (O): Primary outcome was incidence of thyroid cancer (all histologic subtypes). Secondary outcomes included thyroid cancer subtypes or sex-specific associations where reported. (5) Study design (S): Observational studies, including cohort, case-control, and nested case-control studies reporting risk estimates (RR, OR, HR) with 95% confidence intervals (CIs) or sufficient data for calculation. We excluded: studies without a comparison group; studies focusing exclusively on infertility treatments (e.g., assisted reproductive technology) without a clear infertility definition; reviews, editorials, case reports, conference abstracts without sufficient data; studies lacking effect estimates or sufficient data to calculate them.

### 2.2 Literature search, data extraction, and quality assessment

We performed a comprehensive literature search of PubMed, Web of Science, and Scopus from database inception to February 10, 2026. Search terms combined keywords and MeSH terms for “infertility” and “thyroid cancer,” including relevant synonyms (e.g., “subfertility,” “sterility,” “thyroid carcinoma,” “thyroid neoplasm”). No language restrictions were applied. Reference lists of included studies and relevant reviews were screened to identify additional eligible studies. The detailed search strategies for each database are provided in **Supplementary Table 1**.

Two reviewers (XQ and XT) independently screened titles, abstracts, and full texts, with disagreements resolved through discussion or consultation with a third reviewer (JY). Data extracted included author, year, country, study design, sample size, participant characteristics, infertility definition, outcome type, effect estimates, and adjusted confounders. Study quality was assessed using the Newcastle–Ottawa Scale (NOS) (18), which evaluates selection, comparability, and exposure/outcome assessment. Studies scoring ≥7 was considered high quality.

### 2.3 Statistical analysis

Effect estimates were harmonized as RRs for meta-analysis. Pooled RRs and 95% CIs were calculated using random-effects models (DerSimonian–Laird), with fixed-effect models applied for sensitivity analyses. Heterogeneity was assessed using Cochran’s Q test and the *I²* statistic (*I²* >50% indicating substantial heterogeneity) (19). Subgroup analyses were performed by sex and study design. Sensitivity analyses included leave-one-out and influence analyses (20). Publication bias was assessed using funnel plots, Egger’s and Begg’s regression test (21), and the trim-and-fill method (22). To explore potential sources of heterogeneity, we conducted subgroup analyses according to the definition of infertility used in the original studies (reproductive/biological definition, medical record–based diagnosis, self-reported infertility, or not reported). Moreover, a multivariable meta-regression analysis was conducted to examined several factors that could contribute to variability across studies, including sex distribution, study design, geographic region, and the definition of infertility. All analyses were performed using R (version 4.3.0) with the meta and metafor packages. Two-sided *p*-values <0.05 were considered statistically significant.

## 3 Results

### 3.1 Study selection and characteristics

**Figure 1:**
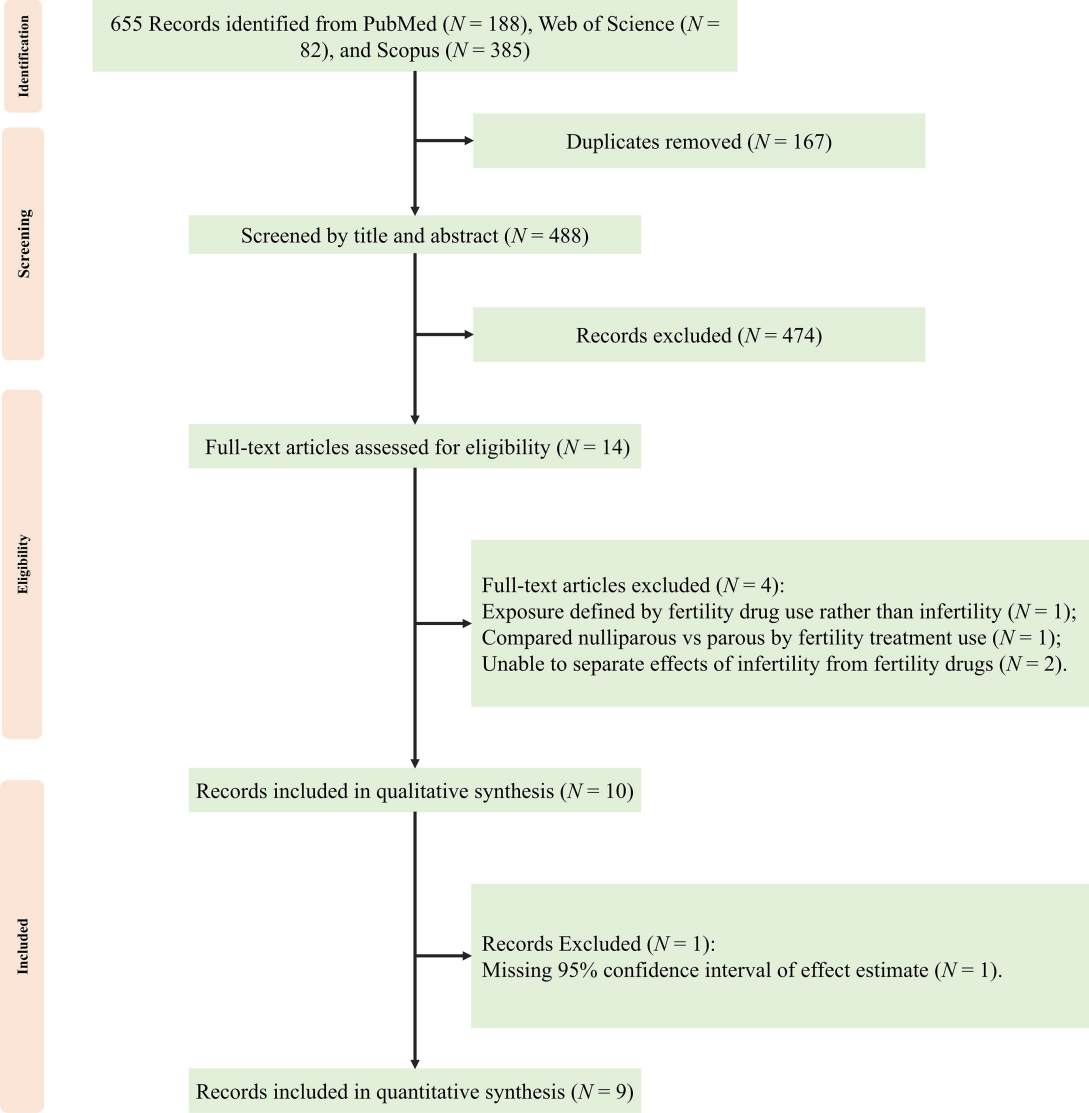
Flow diagram of study selection for the meta-analysis of infertility and thyroid cancer risk. Numbers indicate the studies identified, screened, assessed for eligibility, and included at each stage.

The literature search identified 655 records, of which 9 studies met the inclusion criteria, involving 4,563,090 participants (11–14, 23–27) (**Figure 1**). These included 7 cohort studies (5 retrospective (11, 13, 23, 24, 26) and 2 prospective (12, 25)) and 2 case-control studies (14, 27). Overall, the studies were conducted predominantly in the United States (n = 6) (12, 13, 23, 24, 26, 27), with additional studies from Europe (n = 2) (14, 25) and China (n = 1) (11). Across studies, infertility was defined variably, including clinical diagnosis, self-reported infertility, or exposure to fertility treatment. Most studies adjusted for age and parity (8 of 9 studies) (11–14, 24–27), although adjustment for hormonal factors, autoimmune thyroid disease, and family history was inconsistent (**Table 1**). All included studies were rated as high quality according to the Newcastle–Ottawa Scale (score ≥7; **Tables 2**, **3**).

**Table 1:** Key characteristics of studies included in the meta-analysis of infertility and thyroid cancer (TC) incidence.

Author	Year	Country	Study design	Sample size	Follow up	Age	Infertility type	Infertility diagnosis criteria	Thyroid cancer type	Effect estimate (95% CI)	Covariates adjusted
Ramsay JM, et al (23)	2024	USA	Retrospective cohort	426 azoospermia men and their families, and 3,105 fertile men and their families	51 years	Median 40-49 years	Male infertility (Azoospermia)	sperm concentration of 0 million/ml	All TC (80 cases in infertile group and 396 cases in non-infertile group)	HR 1.54 (1.21-1.97)	/
Wang S, et al (12)	2023	USA	Prospective cohort	26,208 infertile women and 76,872 non-infertile women	infertile women (613,813 person-years) and non-infertile women (1,535,572 person-years)	Median 35 years	Female infertility (28.8% due to ovulatory disorders)	failure to conceive after one year of trying	All TC (142 cases in infertile group and 327 cases in non-infertile group)	HR 1.06 (0.86-1.31)	Age, family history of cancer, race, BMI, age at menarche, oral contraceptive use before age 18, time-varying smoking status, physical activity, AHEI, marital status, recent health seeking behavior, hormonal therapy, gravidity, time-varying gravidity and age at first pregnancy
Murugappan G, et al (24)	2019	USA	Retrospective cohort	64,345 infertile women and 3,128,345 non-infertile women	infertile women (3.8 ± 3.3 years) and non-infertile women (3.9 ± 3.3 years)	infertile group (34.0 ± 5.7 years) and non-infertile group (32.7 ± 7.4 years)	Female infertility	Women were considered to have infertility if they had a diagnosis of infertility (ICD-9: 628.x, 614.6, V26.89; ICD-10: E23.0, N73.6, N97.x, Z31.81), underwent fertility testing (diagnosis codes V26.21, Z31.41), or received fertility treatment, such as a hysterosalpingogram (HSG, CPT 74740).	All TC (138 cases in infertile group and 4869 cases in non-infertile group)	HR 1.29 (1.09-1.53)	Age at index date, index year, nulliparity, race, number of visits per year and highest level of education
Ding DC, et al (11)	2019	China	Retrospective cohort	13,356 infertile women and 53,424 non-infertile women	infertile women (94809 person-years) and non-infertile women (372944 person-years)	infertile group (31.1 ± 5.26 years) and non-infertile group (31.1 ± 5.60 years)	Female infertility	infertility was identified by ICD9: 628.x, 614.6, V26.89	All TC (27 cases in infertile group and 57 cases in non-infertile group)	IRR 1.80 (1.70-1.92)	Age, comorbidity, and medication use
Zamora-Ros R, et al (25)	2015	France	Prospective cohort	345,157 women	infertile women (76,530 person-years) and non-infertile women (1,968,705 person-years)	Mean 51 years	Female infertility	self-report	Differentiated TC (24 cases in infertile group and 327 cases in non-infertile group)	HR 1.70 (1.12-2.60)	Age and country
Eisenberg ML (26),	2015	USA	Retrospective cohort	76083 infertile men and 760830 non-infertile men	infertile men (277,703 person-years) and not report for non-infertile group	infertile group (35.08 ± 5.89 years)	Male infertility	infertility was identified by ICD9: 606.x, V26.21 or by the presence on any claim of a procedure code (CPT) for fertility testing or semen analysis/semen preparation (89300, 89310, 89320, 89321, 89322, 89325, 89329, 89330, 89331).	All TC (27 cases in infertile group and 170 cases in non-infertile group)	HR 1.52 (1.01-2.30)	Age, year of evaluation, comorbidity and follow up time
Brinton LA, et al (13)	2005	USA	Retrospective cohort	8422 infertile women and External control	infertile women (155527 person-years)	Median 30 years	Female infertility	Medical record	All TC (18 cases observed in infertile group and 18.1 cases expected in general population)	SIR 0.99 (0.60-1.60)	Age, race, and calendar year
Negri E, et al (14)	1999	Italy	Case-control	2247 female cases of TC (80% papillary) and 3699 control women	/	Not report	Female infertility	Not report	All TC	OR 1.20 (0.90-1.60)	Age and history of radiation
McTiernan A, et al (27)	1987	USA	Case-control	182 female cases of TC and 389 control women	/	Not report	Female infertility	self-report	All TC	OR 0.81 (0.45-3.10)	Age and weight

**Table 2:** Quality assessment of included cohort studies by Newcastle-Ottawa Scale.

Author	Year	Selection				Comparability		Outcome			Score
		Representativeness of exposed cohort	Selection of non-exposed cohort	Exposure Ascertainment	Outcome present at start of study	Study controls for age	Study controls for any additional important factor	Assessment of Outcome	Length of follow-up	Adequacy of follow-up	
Ramsay JM, et al (23)	2024	★	★	★	★	★		★	★	★	8
Wang S, et al (12)	2023	★	★	★	★	★	★	★	★	★	9
Murugappan G, et al (24)	2019	★	★	★	★	★	★	★	★	★	9
Ding DC, et al (11)	2019	★	★	★	★	★	★	★	★	★	9
Zamora-Ros R, et al (25)	2015	★	★	★	★	★	★	★	★	★	9
Eisenberg ML (26),	2015	★	★	★	★	★	★	★	★	★	9
Brinton LA, et al (13)	2005	★		★	★		★	★	★	★	7

Cohort Studies: Selection ① Representativeness of the exposed cohort★ Selection of the non-exposed cohort★ ② Ascertainment of exposure★ ③ Demonstration that outcome of interest was not present at start of study★. Comparability ① Comparability of cohorts on the basis of the design or analysis★★. Outcome ① Assessment of outcome★ ② Was follow-up long enough for outcomes to occur★ ③ Adequacy of follow up of cohorts★.

**Table 3:** Quality assessment of included case-control studies by Newcastle-Ottawa Scale.

Author	Year	Selection				Comparability		Exposure			Score
		Adequacy of the case definition	Representativeness of cases	Choice of controls	Definition of control	Study controls for age	Study controls for any additional important factor	Exposure assessment	The method of exposure assessment	Non-response rate	
Negri E, et al (14)	1999	★	★	★	★	★	★	★	★		8
McTiernan A, et al (27)	1987	★	★	★	★	★	★	★	★		8

Case-control Studies: Selection ① Adequacy of the case definition★ ② Representativeness of cases★ ③ Choice of controls★ ④ Definition of control★. Comparability ① Comparability of case-controls on the basis of the design or analysis★★. Exposure ① Exposure assessment★ ② The method of exposure assessment★ ③ Non-response rate★.

### 3.2 Association between infertility and thyroid cancer risk

**Figure 2:**
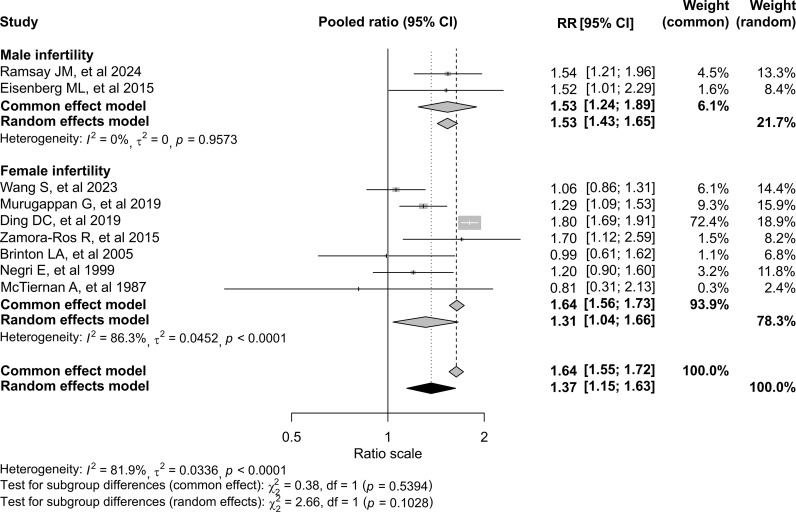
Forest plot of the association between infertility and thyroid cancer risk, stratified by male and female infertility. Diamonds represent pooled estimates from common-effect and random-effects models, with widths indicating 95% confidence intervals (CIs). Heterogeneity within subgroups and overall was assessed using I² and Cochran’s Q. Subgroup differences between male and female infertility were evaluated under both models.

**Figure 3:**
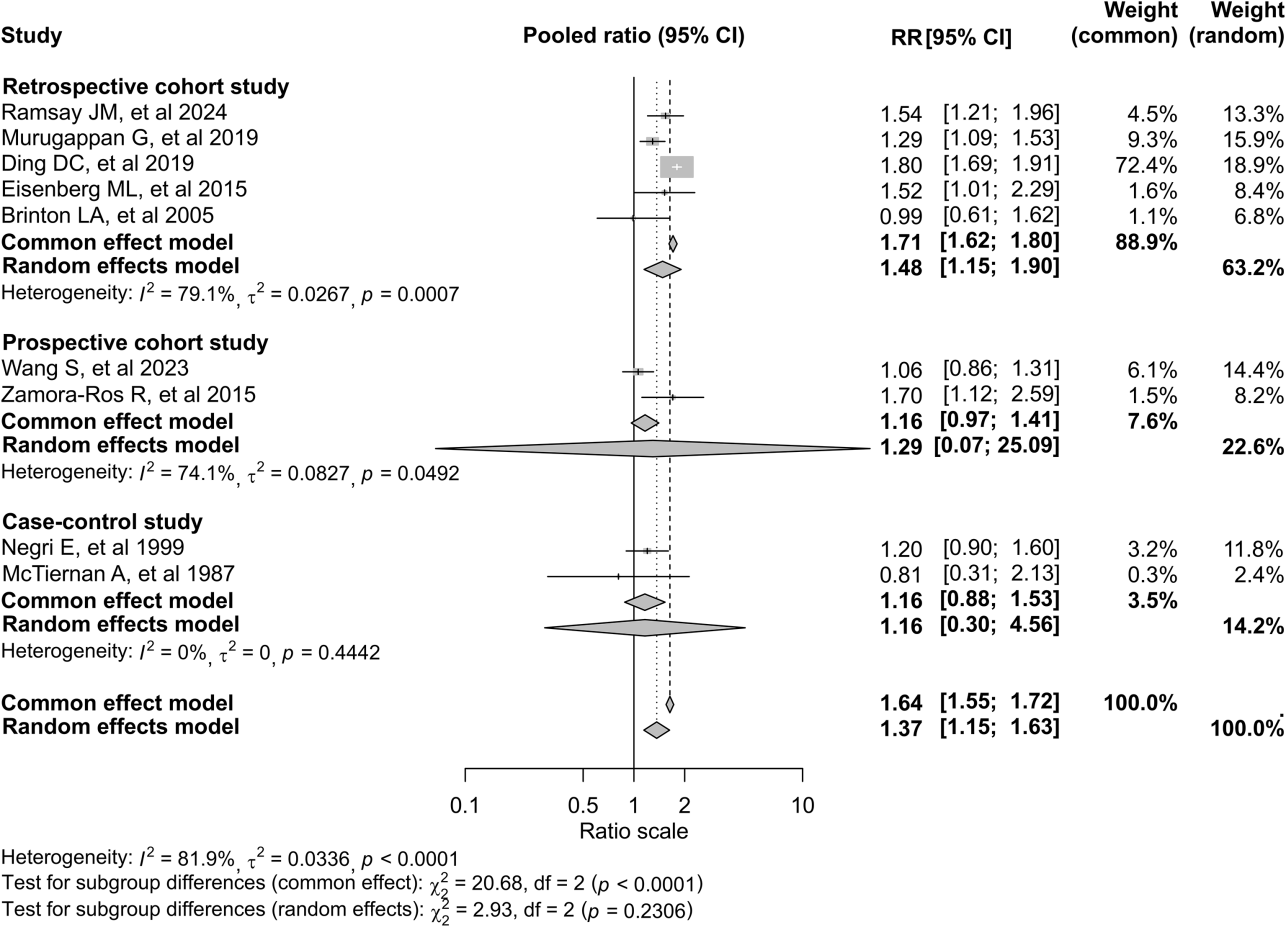
Forest plot of the association between infertility and thyroid cancer risk, stratified by study design. Diamonds indicate pooled estimates from common-effect and random-effects models, with widths representing 95% confidence intervals (CIs). Subgroup and overall heterogeneity were assessed using I² and Cochran’s Q. Differences across study designs were evaluated under both models.

In pooled analyses using a random-effects model, infertility was associated with a 37% increased risk of thyroid cancer (RR, 1.37; 95% CI, 1.15–1.63; **Figure 2**). Between-study heterogeneity was substantial (*I²* = 81.9%; *P* < 0.001). Results were similar under a fixed-effect model. In sex-stratified analyses, the association appeared stronger among men (RR, 1.53; 95% CI, 1.43–1.65) than among women (RR, 1.31; 95% CI, 1.04–1.66), although the test for interaction was not statistically significant (**Figure 2**). When stratified by study design, retrospective cohort studies yielded a pooled RR of 1.48 (95% CI, 1.15–1.90). The two prospective cohort studies showed an imprecise estimate (RR, 1.29; 95% CI, 0.07–25.09). Case-control studies demonstrated a pooled OR of 1.16 (95% CI, 0.30–4.56), with wide confidence intervals and no statistically significant association (**Figure 3**). Subgroup analyses according to infertility definition are presented in **Supplementary Figure 1**. Although effect sizes varied slightly across categories, the direction of the association was broadly consistent. The test for subgroup differences was not statistically significant under the random-effects model (*p* = 0.7994).

### 3.3 Sensitivity analyses and publication bias

**Figure 4:**
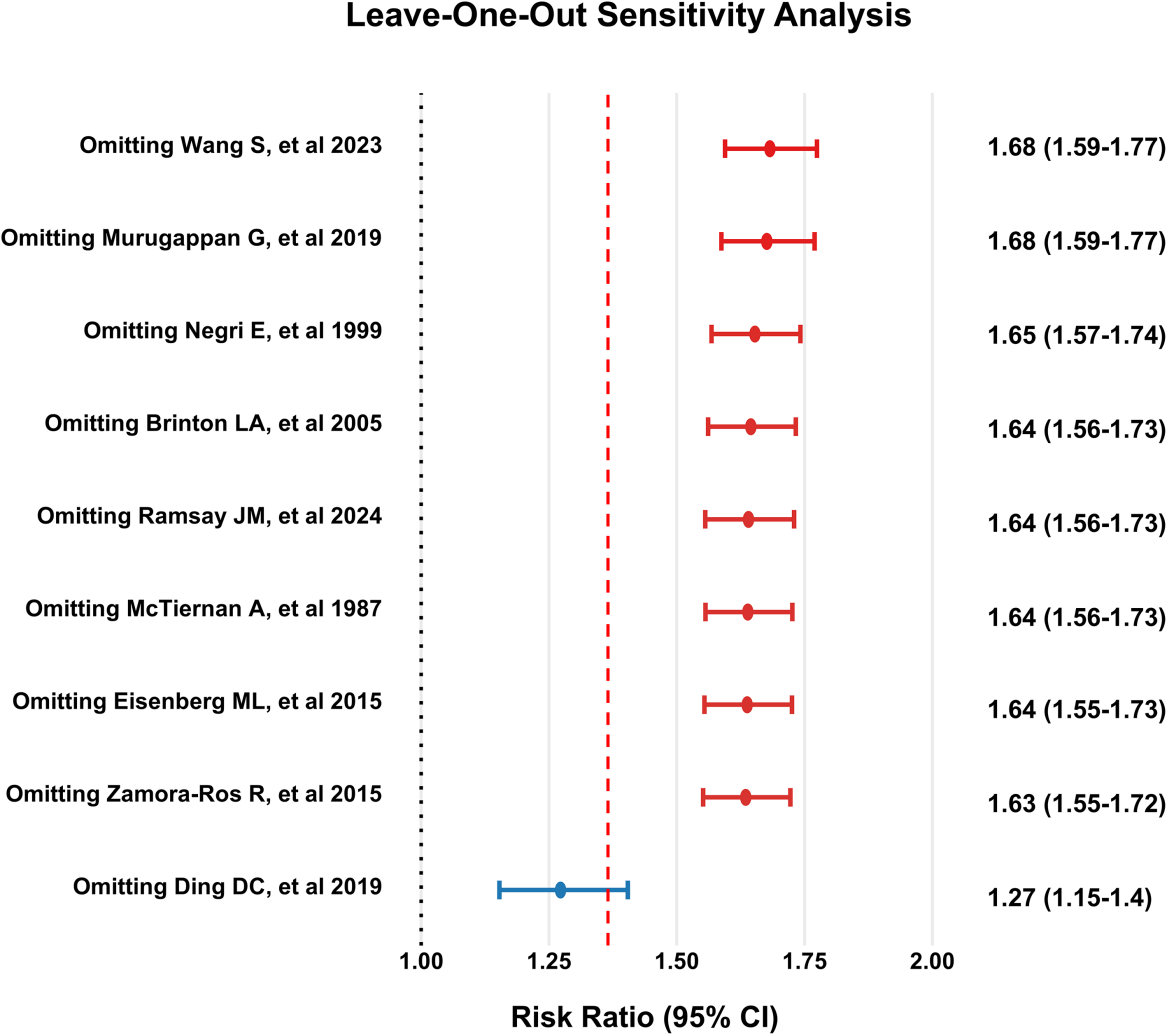
Leave-one-out sensitivity analysis of infertility and thyroid cancer risk. Each line shows the 95% confidence interval (CI) of the pooled risk ratio (RR) after excluding the study on the y-axis, with points representing the pooled RR. The red dashed line shows the pooled RR including all studies, and the black dotted line indicates RR = 1.

**Figure 5:**
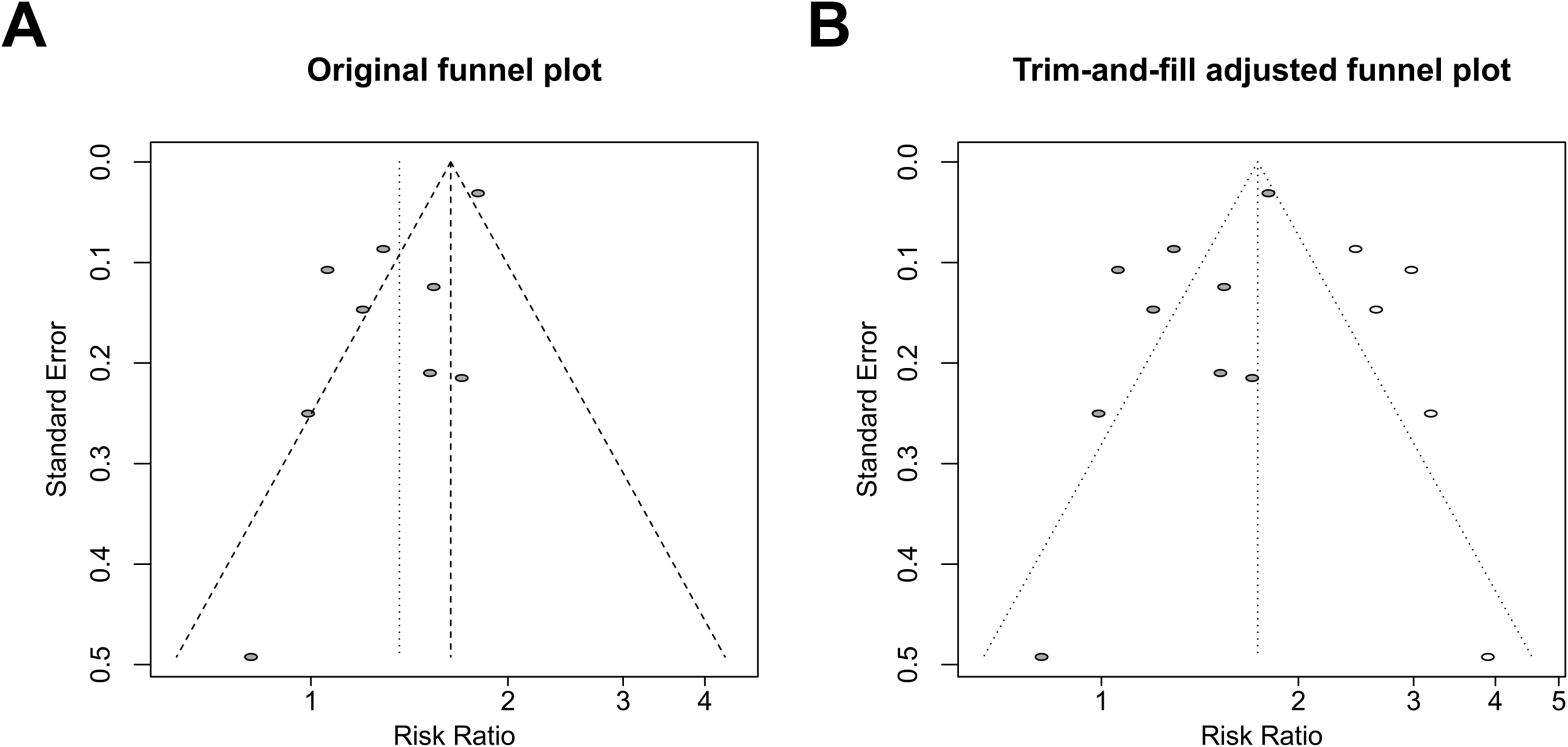
Funnel plots evaluating publication bias in the meta-analysis of infertility and thyroid cancer risk. The left panel shows study-specific risk ratios (RRs) plotted against their standard errors **(A)**, and the right panel shows the trim-and-fill–adjusted plot **(B)**. The vertical dashed line indicates the pooled effect, and diagonal lines represent pseudo 95% confidence limits. The trim-and-fill method assesses the potential impact of small-study effects and publication bias.

Leave-one-out analyses indicated that exclusion of any single study did not materially alter the pooled estimate (RR range, 1.27–1.68), supporting the robustness of the primary findings (**Figure 4**). Visual inspection of the funnel plot suggested possible asymmetry (**Figure 5A**). Egger’s regression test indicated evidence of small-study effects (*t* = −2.83; *P* = 0.026), whereas Begg’s test did not detect significant asymmetry (*z* = 0.42; *P* = 0.68). Using the trim-and-fill method, five potentially missing studies were imputed (**Figure 5B**). The adjusted pooled estimate remained statistically significant (RR, 1.73; 95% CI, 1.35–2.22; *P* = 0.0004). However, between-study heterogeneity remained substantial (*I²* = 87.7%; 95% CI, 81.1%–92.0%).

### 3.4 Meta-regression

Female infertility was not significantly associated with thyroid cancer risk compared with male infertility (β = −0.1923, 95% CI: −3.0544 to 2.6698; *P* = 0.5501; **Supplementary Table 2**). No significant effect modification was observed by geographic region, with studies from East Asia (β = 0.3614, 95% CI: −0.7496 to 1.4724; *P* = 0.1511) and West Europe (β = 0.4327, 95% CI: −3.1695 to 4.0349; *P* = 0.6920) showing no differences compared with North America. Similarly, study design did not influence the association, as prospective cohort (β = −0.1812, 95% CI: −3.7234 to 3.3611; *P* = 0.6331) and case-control studies (β = −0.4898, 95% CI: −5.3543 to 3.3746; *P* = 0.4223) were comparable to retrospective cohorts. The definition of infertility was also not associated with differences in effect estimates, with medical record–based and self-reported measures yielding similar results to reproductive/biological definitions. Overall, these findings indicate that sex, region, study design, and infertility definition did not significantly explain between-study heterogeneity.

## 4 Discussion

In this systematic review and meta-analysis of nine observational studies including over 4.5 million participants, we found that infertility was associated with a 37% increased risk of thyroid cancer. Although between-study heterogeneity was substantial, the positive association persisted across sensitivity analyses and remained statistically significant after adjustment for potential small-study effects. Our pooled estimate helps contextualize and reconcile the heterogeneous findings reported by individual studies. Several large cohort studies have suggested an elevated thyroid cancer risk among infertile individuals (11, 23–26), whereas others reported null or modest associations (12–14, 27). By synthesizing available evidence, our analysis provides a more precise and stable estimate, suggesting that infertility itself—rather than isolated findings in specific populations—may be associated with increased thyroid cancer incidence. Importantly, this association persisted despite substantial heterogeneity and evidence of small-study effects. The trim-and-fill–adjusted estimate remained statistically significant and even strengthened, indicating that the observed association is unlikely to be entirely explained by publication bias.

Our results extend prior literature by focusing specifically on infertility rather than fertility treatment exposure alone. Previous meta-analyses primarily evaluated fertility drugs—particularly clomiphene citrate—and reported modest increases in thyroid cancer risk (15). However, by separating infertility as the exposure of interest, our analysis provides a clearer estimate of its independent association with thyroid cancer. This distinction is important, as infertility itself may reflect underlying endocrine, autoimmune, or metabolic dysregulation that could plausibly influence thyroid carcinogenesis independent of pharmacologic stimulation (10).

Infertility encompasses a heterogeneous group of conditions with distinct etiologies in men and women. Female infertility may result from ovulatory disorders, tubal obstruction, uterine abnormalities, or endometriosis, whereas male infertility is often related to impaired spermatogenesis, hormonal disturbances, or genetic factors. These differences may contribute to heterogeneity when both sexes are analyzed together. In sex-stratified analyses, the association between infertility and thyroid cancer appeared stronger among men than among women, although the interaction test was not statistically significant. This finding should be interpreted with caution but warrants discussion. Male infertility often reflects more profound endocrine dysfunction, including abnormalities in the hypothalamic–pituitary–gonadal axis, altered androgen-to-estrogen balance, and increased peripheral aromatization of testosterone to estradiol (28, 29). Estrogen signaling has been shown to promote thyroid cell proliferation and tumor progression, and relative estrogen excess in men with impaired androgen production may therefore confer disproportionate thyroid cancer susceptibility (30). In addition, infertile men undergoing clinical evaluation may represent a more selected subgroup with severe reproductive or systemic abnormalities, potentially inflating relative risk estimates (31). Differences in healthcare utilization may also contribute: infertility-related medical contact may represent a larger relative increase in thyroid surveillance among men than among women, in whom gynecologic care is already routine (32). Finally, male-specific estimates were derived from a limited number of studies and cases, increasing vulnerability to random error and residual confounding (23, 26). Taken together, the observed sex difference should be considered hypothesis-generating rather than definitive and underscores the need for adequately powered, sex-stratified prospective studies.

Substantial heterogeneity was observed across included studies, reflecting differences in study design, infertility definitions, comparator groups, and covariate adjustment. Retrospective cohort studies yielded stronger associations than prospective cohorts and case–control studies. This pattern raises the possibility of detection bias, as infertile individuals often undergo more frequent medical evaluations, increasing the likelihood of incidental thyroid cancer detection (11). Prospective cohorts, although methodologically stronger for temporal inference, were few and produced imprecise estimates due to sparse outcome events (33, 34). Variation in infertility definitions likely contributed further to heterogeneity (16). Some studies relied on clinical diagnoses, others on self-reported infertility or fertility treatment exposure, and comparison groups ranged from the general population to non-infertile individuals. Such heterogeneity complicates interpretation and may obscure distinctions between infertility-related biological susceptibility and treatment-related effects (16). Residual confounding remains a key concern. While most studies adjusted for age and parity, fewer accounted for important factors such as body mass index, smoking, autoimmune thyroid disease, family history of thyroid cancer, or detailed hormonal profiles. Autoimmune thyroid disease is more prevalent among infertile individuals and may independently increase thyroid cancer risk (35, 36). Incomplete control for these factors may partially explain observed associations. Surveillance bias is another important consideration. Individuals with infertility often have more frequent healthcare contact, including laboratory testing and imaging, compared with the general population. Surveillance bias has been recognized as a factor that can misinterpret differences in cancer risk when healthcare utilization differs between groups, and enhanced detection has been implicated in the rising incidence of thyroid cancer through the identification of small, asymptomatic tumors (37). However, the elevated risks persisting beyond early follow-up periods, suggesting that surveillance bias alone is unlikely to fully account for the association. Nonetheless, future studies should incorporate analytic strategies to better address detection bias, such as lag-time analyses and restriction to clinically significant cancers.

Additionally, we conducted an additional subgroup analysis according to the method used to ascertain infertility, including reproductive/biological definitions, medical record–based diagnoses, self-reported infertility, and studies without a clearly reported definition. Although effect estimates varied slightly across categories, the overall direction of association remained broadly consistent. Moreover, the test for subgroup differences was not statistically significant under the random-effects model, suggesting that differences in infertility definitions alone are unlikely to fully explain the observed heterogeneity. Furthermore, some causes, such as ovulatory disorders and polycystic ovary syndrome, are strongly associated with hormonal dysregulation, whereas others—such as tubal obstruction or certain anatomical abnormalities—may occur independently of endocrine disturbances. The inclusion of heterogeneous infertility etiologies in observational studies may therefore dilute associations driven primarily by endocrine-mediated mechanisms. Because most studies included in our meta-analysis did not report detailed infertility etiologies, etiology-specific analyses were not feasible. Future research with more detailed clinical characterization of infertility causes will be important to clarify whether endocrine-related infertility subtypes are differentially associated with thyroid cancer risk.

The current study provides comprehensive evidence on the association between infertility and thyroid cancer risk and systematically evaluates potential sources of between-study heterogeneity. Multivariable meta-regression indicated that key study-level characteristics—including sex, study design, geographic region, and infertility definition—did not materially influence the observed association, suggesting that the findings are broadly consistent across diverse settings and methodological approaches. However, the potential for detection bias warrants careful consideration. Individuals undergoing infertility evaluation are more likely to have frequent healthcare interactions and diagnostic imaging, which may increase incidental detection of thyroid cancer independent of true biological risk. Although this could not be formally assessed in the present analysis, it remains a plausible explanation for part of the observed association. Future studies should prioritize designs that explicitly address detection bias, such as incorporating lag-time analyses or adjusting for healthcare utilization. In addition, more rigorous control of key confounders—including obesity, smoking, and reproductive hormone exposure—is essential to disentangle underlying mechanisms. Together, these considerations highlight the need for methodologically robust, prospective studies to clarify the causal nature and clinical implications of the infertility–thyroid cancer relationship.

This study has several strengths. We adhered to PRISMA guidelines, conducted a comprehensive literature search across multiple databases, included high-quality studies, and performed extensive subgroup, sensitivity, and publication bias analyses. The large pooled sample size enhanced statistical power and enabled exploration of sex-specific associations. However, limitations should be acknowledged. All included studies were observational, precluding causal inference. Exposure misclassification was likely due to heterogeneous infertility definitions and limited information on infertility duration, etiology, and treatment history. Evidence of small-study effects was detected, although adjusted analyses supported the robustness of the association. Finally, the limited number of prospective studies restricted our ability to assess temporality and dose–response relationships. While a stronger association between infertility and thyroid cancer risk was observed in men, this finding should be interpreted with caution due to the limited number of male-specific studies and the non-significant interaction test. Given the current evidence, it would be premature to draw definitive conclusions about the role of biological sex in this relationship. Additionally, male infertility may act as a marker for broader endocrine or genetic dysregulation (38), but the small number of studies and the potential for inflated precision estimates due to sparse data must be considered.

Given the rising prevalence of infertility and the increasing incidence of thyroid cancer, clinicians should be aware of the possible increased thyroid cancer risk among infertile individuals, particularly among individuals with additional endocrine or autoimmune risk factors. Future research should prioritize large, well-designed prospective cohort studies with standardized infertility definitions, detailed characterization of infertility etiology, hormonal and autoimmune profiling, and rigorous control for surveillance bias. Such studies are essential to clarify causality, identify high-risk subgroups, and disentangle the roles of infertility biology and fertility treatments in thyroid carcinogenesis.

Both infertility and thyroid cancer have profound implications for patients’ quality of life, psychosocial well-being, and long-term survivorship (39, 40). While epidemiologic risk estimation is crucial, it is equally important to consider how these conditions affect individuals’ daily lives. Patient-reported outcomes (PROs) have emerged as essential tools in assessing the broader clinical significance of reproductive and oncologic conditions (41, 42). PROs provide valuable insights into the impact of disease and treatment on patients’ physical, emotional, and social well-being, offering a more comprehensive understanding of the burden these conditions impose. We recommend further investigation into the role of PROs in this context, as highlighted in recent studies, which underscores the importance of PROs in oncology clinical trials and healthcare decision-making (43). Future studies should consider incorporating PRO assessments to better contextualize the translational relevance of infertility and thyroid cancer epidemiology.

## 5 Conclusion

This study suggests an association between infertility and increased thyroid cancer risk, with consistent findings across sensitivity analyses. However, observational studies cannot establish causality. Residual confounding from factors like endocrine disorders, socioeconomic status, and treatment exposures, as well as reverse causation, remain plausible. Infertility may act as a surrogate marker of shared hormonal or metabolic pathways rather than a direct cause of thyroid cancer. Further research is needed to clarify these potential mechanisms.

## Data Availability

The original contributions presented in the study are included in the article/**Supplementary Material**. Further inquiries can be directed to the corresponding authors.
