# Supplementary material for: Association between infertility and thyroid cancer risk: a systematic review and meta-analysis
Source: Front Oncol. 2026 May 18;16:1813942. doi: 10.3389/fonc.2026.1813942 (PMC13222791; doi:10.3389/fonc.2026.1813942)
Supplement: Supplementary file 1 [file DataSheet1.pdf]

Supplementary Table S1. Literature Search Strategy

Databases	Search strategy
PubMed	("Infertility"[Mesh] OR infertility OR subfertility OR sterility OR "female infertility" OR "reproductive dysfunction") AND ("Thyroid Neoplasms"[Mesh] OR "thyroid cancer" OR "thyroid carcinoma" OR "thyroid malignancy" OR "papillary thyroid carcinoma" OR "follicular thyroid carcinoma" OR "medullary thyroid carcinoma" OR "anaplastic thyroid carcinoma")
Scopus	TITLE-ABS-KEY("infertility" OR "subfertility" OR "sterility" OR "female infertility" OR "reproductive dysfunction") AND TITLE-ABS-KEY("thyroid cancer" OR "thyroid carcinoma" OR "thyroid neoplasm*" OR "thyroid malignancy" OR "papillary thyroid carcinoma" OR "follicular thyroid carcinoma" OR "medullary thyroid carcinoma" OR "anaplastic thyroid carcinoma")
Web of Science	TS=("infertility" OR "subfertility" OR "sterility" OR "female infertility" OR "reproductive dysfunction") AND TS=("thyroid cancer" OR "thyroid carcinoma" OR "thyroid neoplasm*" OR "thyroid malignancy" OR "papillary thyroid carcinoma" OR "follicular thyroid carcinoma" OR "medullary thyroid carcinoma" OR "anaplastic thyroid carcinoma")

Supplementary Table S2. Multivariable meta-regression analysis of the association between infertility and thyroid cancer risk

Characteristic	Association between infertility and thyroid cancer risk		
	**Meta-regression coefficient (95% CI)**	***P* value**	R^2
**Intercept**	0.4318 (-1.1478 to 2.0114)	0.1785	
**Study design**			0.047
Retrospective cohort	Reference	/	
Prospective cohort	-0.1812 (-3.7234 to 3.361)	0.6331	
Case-control	-0.4898 (-5.3543 to 4.3746)	0.4223	
**Sex**			-0.138
Male	Reference	/	
Female	-0.1923 (-3.0544 to 2.6698)	0.5501	
**Region**			0.558
North America	Reference	/	
West Europe	0.4327 (-3.1695 to 4.0349)	0.3692	
East Asia	0.3614 (-0.7496 to 1.4724)	0.1511	
**Infertility definition**			-0.399
Reproductive/biological definition	Reference	/	
Medical record–based diagnosis	-0.0131 (-3.1128 to 3.0866)	0.9659	
Self-reported	0.0397 (-3.5625 to 3.6419)	0.9115	

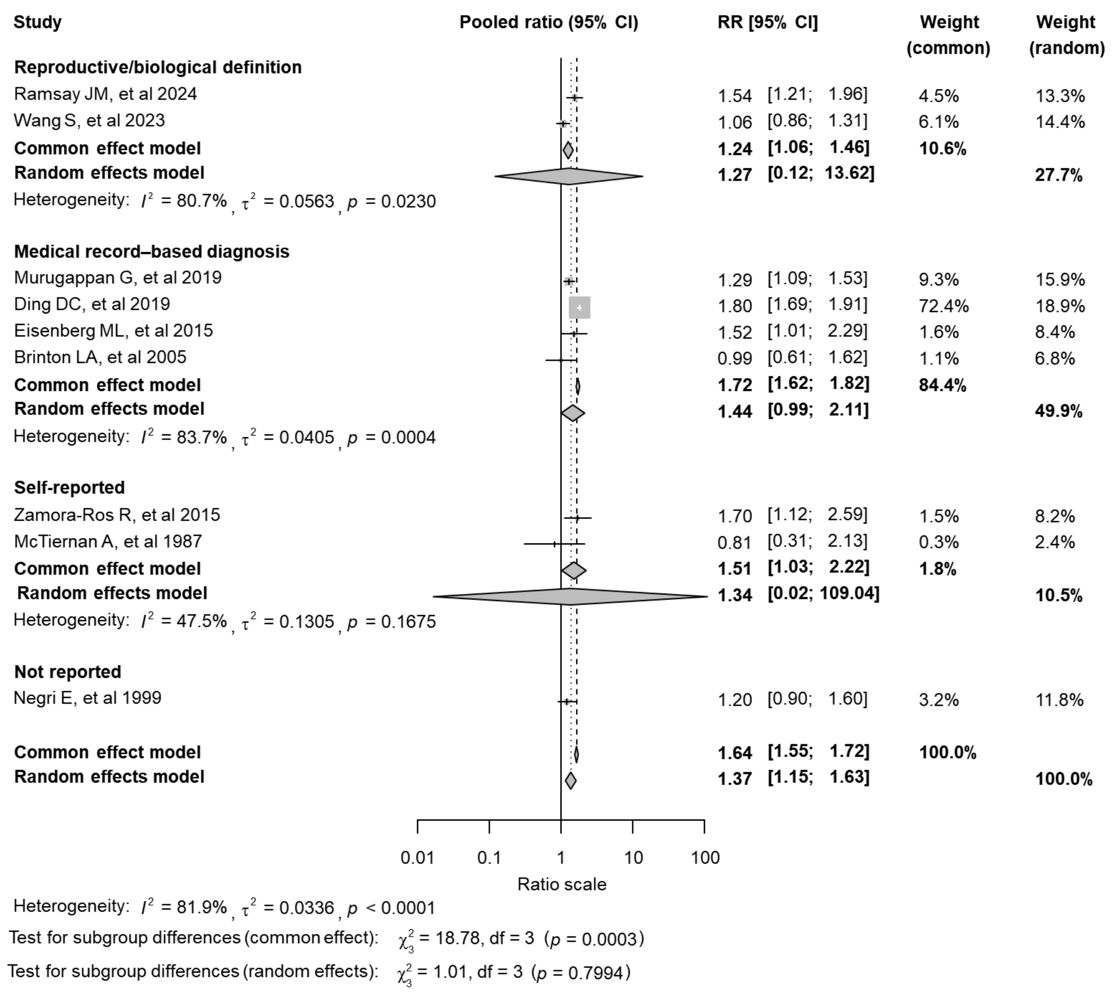

Supplementary Figure S1. Forest plot of the association between infertility and thyroid cancer risk, stratified by infertility definition. Squares represent study-specific effect estimates, with horizontal lines indicating 95% confidence intervals (CIs). Diamonds represent pooled estimates from the common-effect and random-effects models, with widths corresponding to the 95% CIs. Between-study heterogeneity within subgroups and overall was assessed using Cochran's Q test and the I^2 statistic. Differences between subgroups defined by infertility ascertainment were evaluated under both models.
